# Pachychoroid Diseases of the Macula

**Published:** 2014

**Authors:** Roberto Gallego-Pinazo, Rosa Dolz-Marco, Francisco Gómez-Ulla, Sarah Mrejen, K Bailey Freund

**Affiliations:** 1 Unit of Macula, Department of Ophthalmology, University and Polytechnic Hospital La Fe, Valencia, Spain; 2 RETICS Oftared, Health Institute Carlos III, Madrid, Spain; 3 Department of Ophthalmology, University of Santiago de Compostela, Santiago de Compostela, Spain; 4 Ophthalmic Institute Gomez-Ulla, Santiago de Compostela, Spain; 5 Vitreous, Retina, Macula Consultants of New York, New York, USA; 6 LuEsther T. Mertz Retinal Research Center, Manhattan Eye, Ear, and Throat Hospital, New York, USA; 7 Department of Ophthalmology, New York University School of Medicine, New York, USA

**Keywords:** Pachychoroid Diseases, Macula, Central serous chorioretinopathy

## Abstract

Advances in optical coherence tomography have enabled a better appreciation of the role of pathologic choroidal changes in a variety of retinal disease. A “pachychoroid” (pachy-[prefix]: thick) is defined as an abnormal and permanent increase in choroidal thickness often showing dilated choroidal vessels and other structural alterations of the normal choroidal architecture. Central serous chorioretinopathy is just one of several pachychoroid-related macular disorders. This review summarizes the current state of knowledge of the pachycoroid spectrum and the hallmark features seen with multimodal imaging analysis of these entities

## INTRODUCTION

Rapid progress in retinal imaging has provided new insights into a variety of chorioretinal disorders. Advances in optical coherence tomography (OCT) are perfect examples of this trend. Following the first descriptions of retinal OCT imaging (1–3), the subsequent evolution led to improvements in resolution to better visualize the retinal structure (4–6). More recently, further advances in choroidal imaging occurred. Coming on of enhanced depth imaging (EDI) (7) and swept source OCT technologies (8) have enabled more precise analysis of the choroid both qualitatively and quantitatively.

Three different layers of choroidal tissue may be differentiated between Bruch’s membrane and the sclera-choroidal junction: choriocapillaris is the innermost portion; Sattler’s layer, composed of small oval vascular patterns, and Haller’s layer consisting of large outer oval vascular patterns (9). This qualitative analysis is rough, and in the absence of pathologic changes all three layers may be found in a high-penetration OCT (both EDI and SS). On the other hand, the quantitative analysis of choroidal thickness is more variable. Choroidal thickness decreases with increasing age and axial length (10–12). Gender is not associated with differences in choroidal thickness (13). Transiently increased choroidal thickness was associated with acute stages of several posterior uveitis such as Vogt-Koyanagi-Harada disease (14), multifocal choroiditis (15) and the multiple evanescent white dot syndrome (16).

**Figure 1.**
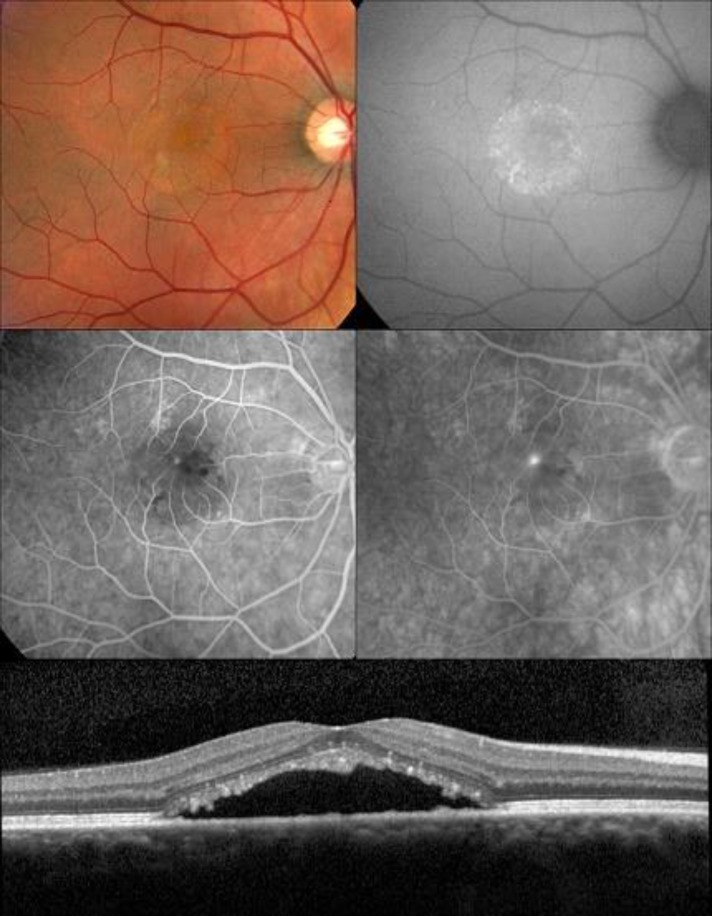
Forty year-old man with typical CSC

The term pachychoroid (pachy-[prefix]: thick) was proposed as a term indicating an abnormal and permanent increase in choroidal thickness, This entity often occurs in eyes with central serous chorioretinopathy (CSC) or CSC-like features (17–19). Eyes with a pachychoroid change often manifest dilatation of the large choroidal vessels compressing the overlying choriocapillaris and Sattler’s layer (19). The aim of the present review is to define the morphological characteristics of the pachychoroid occurring in CSC and to analyze the new spectrum of diseases sharing similar choroidal findings (Fig. 1).

*Central Serous Chorioretinopathy*

Central serous chorioretinopathy is characterized by the presence of subretinal fluid often associated with serous pigment epithelial detachment (PED). Abnormalities of the choroidal circulation are believed to play an important role in the pathophysiology of this entity. Choroidal congestion and hyperpermeability have been frequently described in this disease (20–22). The correlate of such findings on OCT imaging is increased choroidal thickness (pachychoroid), particularly involving Haller’s layer, as the unifying feature of all types of CSC (23,24). The chronic presence of fluid may manifest as focal areas of speckled hyperautofluorescence and eventually to gravitational zones corresponding to serous retinal detachments (Fig. 1) (24,25).

*Pachychoroid Pigment Epitheliopathy*

Pachychoroid pigment epitheliopathy (PPE) is a newly described entity with characteristic features on multimodal imaging of the macula (18). This entity is distinguished from typical CSC as the patients manifesting with retinal pigment epithelium (RPE) but had no documented subretinal fluid. Pachychoroid pigment epitheliopathy may represent a *formae frustae* of CSC, similar in nature to the findings often seen in the fellow eye of patients presenting with unilateral acute CSC.

**Figure 2:**
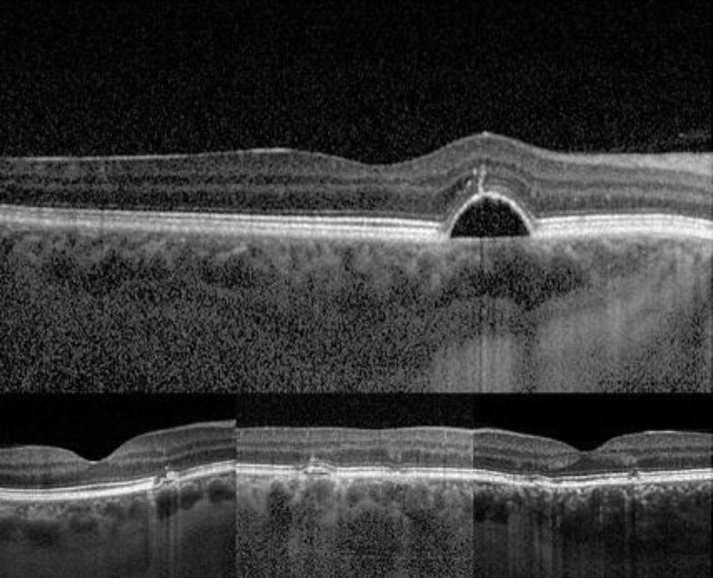
Morphologic changes identified in the OCT images of cases with PPE

- Fundus examination often shows an orange-reddish appearance and absence of normal tessellation what indicates an underlying pachychoroid. Present changes in pigment include: RPE alterations that might be mistaken for age-related maculopathy (ARM), a pattern dystrophy of the RPE or non-neovascular age-related macular degeneration (AMD). These changes may include sub-RPE deposits similar in appearance to typical drusen.
- OCT imaging shows numerous, scattered small elevations of the RPE representing RPE hyperplasia and sub-RPE drusen-like deposits (Fig. 2). Occasionally, small serous PEDs are present. The defining feature of PPE on OCT is a thick choroid usually located directly beneath the clinically apparent RPE change. Large choroidal vessels in the Haller’s layer often approximate Bruch’s membrane without superseding Sattler’s layer (Fig. 2).
- Indocyanine green angiography (ICGA) reveals choroidal hyperpermability as mid-phase hyperfluorescence that co-existent with the areas of RPE disturbances.
- Fundus autofluorescence shows areas of granular hypoatuofluorescence and mixed stippled hyper- and hypoautofluorescence. Signs of antecedent subretinal fluid, such as gravitational tracts, zonal areas of hyperautofluorescence or focal areas of speckled hyperautofluorescence are never seen in PPE.

*Pachychoroid Neovasculopathy*

Pachychoroid neovasculopathy (PNV) is considered a late complication of PPE and chronic CSC in patients who presumably carry a genetic risk for choroidal neovascularization (19). The unique imaging findings include features similar to those described for PPE and CSC, but eyes with PNV also show evidence of sub-RPE neovascular tissue -type 1 neovascularization- (26). Pachychoroid neovasculopathy may be discovered as an incidental finding in patients with a history of CSC, with or without associated subretinal fluid collections. Patients lacking a history of CSC may present with symptoms of an exudative maculopathy related to this slow-growing form of choroidal neovascularization. The current hypothesis suggests that in susceptible individuals, long-term effects on the RPE, Bruch’s membrane and choriocapillaris induced by a pachychoroid may lead to the subsequent development of type 1 neovascularization (27,28).

**Figure 3:**
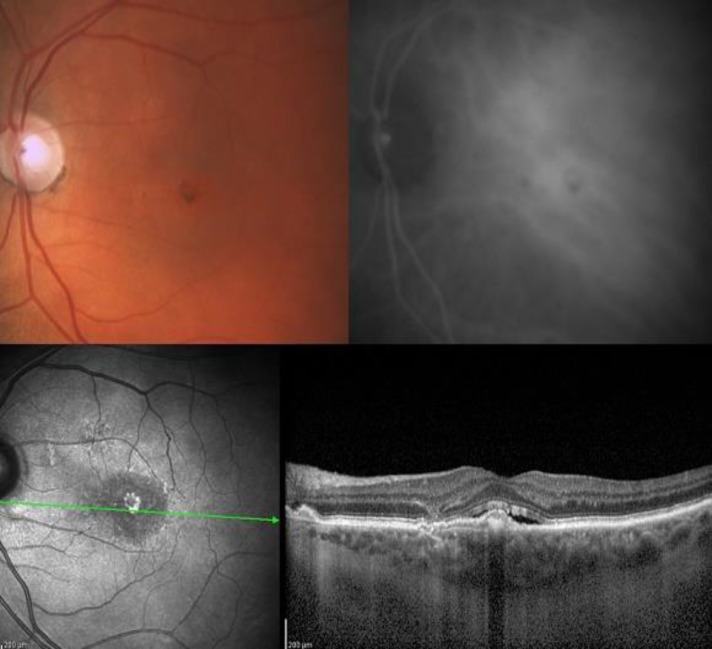
Fifty-three-year-old man with typical changes of pachychoroid neovasculopathy

Optical coherence tomography imaging in PNV shows a broad shallow elevation of the RPE representing neovascular proliferation within Bruch’s membrane (26). This form of type 1 neovascularization is typically found overlying an area of localized choroidal thickening with dilated choroidal vessels (Fig. 3).

Autofluorescence image (top right) shows mottling hyperautofluorescence. Fluorescein angiography identifies the juxtafoveal leaking point (middle row). OCT showing subretinal fluid with photoreceptor outer segment elongation (bottom row).

Small elevation of the retinal pigment epithealium (top row), drusen-like deposits underneath the retinal pigment epithelium (bottom row). All cases show large choroidal vessels within choroidal Haller’s layer approaching Bruch’s membrane with the absence of an intervening Sattler’s layer.

Color photographed evidenced orange-reddish appearance and absence of standard tessellation (top left) Fundus autofluorescence image showed focal changes of granular hypoatuofluorescence and mixed stippled hyper and hypoautofluorescence (top right). Indocyanine green angiography revealed a significant choroidal hyperpermeability (bottom left). OCT evidenced a broad shallow elevation of the retinal pigment epithelium representing a type 1 neovascular proliferation with associated flat subretinal fluid (bottom right).

Indocyanine green angiography often shows both mid-phase patchy areas of choroidal hyperpermability and a discrete plaque of late hyperfluorescence corresponding to type 1 neovascular tissue.

Eventually, polypoidal choroidal vasculopathy (PCV) may develop, within or at the margins of the slow-growing type 1 neovascular tissue. The polyps may produce an exudative maculopathy that includes both lipid deposition and hemorrhagic complications. The polyps are easily identified with ICGA as early focal areas of intense hyperfluorescence that may show either late leakage or “wash-out” appearance depending on their degree of activity.

## CONCLUSION

Enhanced depth imaging and SS OCT have enabled a better appreciation of the role of pathologic choroidal changes in CSC. Furthermore, they have helped defining an expanded spectrum of pachychoroid-related macular disorders. Recognition of these entities is important as they may mimic other diagnoses that have different natural courses and treatments. For example, the clinical appearance of PPE may simulate ARM, AMD, or pattern dystrophies. Similarly, without choroidal imaging, PNV may easily be mistaken for neovascular AMD.

The occurrence of PCV in eyes with PNV adds further evidence that PCV is a natural evolution of type 1 neovascular proliferation rather than a disease *sui generis*.

In our experience, eyes with exudation due to PNV, irrespective of the presence of PCV, are more resistant to anti-vascular endothelial growth factor (anti-VEGF) agents than eyes with choroidal neovascularization (CNV). This is presumably due to the typical AMD and myopic macular degeneration. Thus, the recognition of an underlying pachychoroid in neovascularized maculo- pathies may have important implications regarding their management. Since the photodynamic therapy is intended to reduce choroidal thickness and hyperpermeability, it surely warrants further exploration (29,30).
